# Integrated Photodetectors Based on Group IV and Colloidal Semiconductors: Current State of Affairs

**DOI:** 10.3390/mi11090842

**Published:** 2020-09-08

**Authors:** Principia Dardano, Maria Antonietta Ferrara

**Affiliations:** National Research Council (CNR), Institute of Applied Sciences and Intelligent Systems, Via Pietro Castellino 111, 80131 Naples, Italy

**Keywords:** photodetector, semiconductor, microphotonics, group IV, colloidal systems

## Abstract

With the aim to take advantage from the existing technologies in microelectronics, photodetectors should be realized with materials compatible with them ensuring, at the same time, good performance. Although great efforts are made to search for new materials that can enhance performance, photodetector (PD) based on them results often expensive and difficult to integrate with standard technologies for microelectronics. For this reason, the group IV semiconductors, which are currently the main materials for electronic and optoelectronic devices fabrication, are here reviewed for their applications in light sensing. Moreover, as new materials compatible with existing manufacturing technologies, PD based on colloidal semiconductor are revised. This work is particularly focused on developments in this area over the past 5–10 years, thus drawing a line for future research.

## 1. Introduction

Microphotonics is a branch of microelectronics where devices manipulates light on a microscopic scale and it is used in optical networking. Essentially, it consists in wafer-level integrated devices and systems that allow to emit, transmit, detect, and process light along with other forms of radiant energy with photon as the quantum unit [1]. The immunity of optical signals to external influences, such as the intensification of light beams without interaction and the possibility to realize cheap densely packed components capable of large-scale integration, make microphotonics an area of great interests in research and development [2].

In order to exploit the existing technology in microelectronics, Silicon (Si) is considered the material to be investigated to realize integrated photonic circuits. For this reason, silicon-based microphotonics has gained growing interest over the past decades. Indeed, fabrication technologies employed for micro- and nano-silicon photonics allow the integration of electronic, photonic, and sensing devices on the same chip with a very low cost. In recent decades, considerable efforts have been devoted to the development of new silicon photonic components that have led to innovative solutions with applications in the field of telecommunications and multichip optical interconnections and which promise to improve the performance of the next generation of commercial processors.

Such efforts have given rise, for example, to new complementary metal-oxide-semiconductor (CMOS) configurations of optical modulators capable of operating in the gigahertz regime, waveguides and photonic crystal switches (PhC) to direct the light on chips on a sub-micrometric scale [3]. These include the innovative use of nanostructures with a negative effective refractive index capable of guiding light with very low losses [4,5] and switching its optical path through unprecedented effects [6]. Furthermore, the matching of processing technologies have paved the way for new devices integrated with fluidic, electronic, and photonic circuits for sensing detection and healthcare, which have led to the development of a new generation of biochips to be applied in fields ranging from biomedical to environmental [7,8,9].

On the other hand, for a complete manipulation of the light on a single intelligent microsystem, close integration with an emissive source and a detector capable of receiving the modulated light and switching it into an electrical signal is crucial. In this direction, new experiments on Raman emission in silicon nanocrystals have shown an extremely high increase of the optical gain [10,11,12], encouraging the research on this way.

Finally, to complete a perfectly integrated optoelectronic multisystem, it is necessary to comply with the need to detect light and convert it into an electrical signal, all using semiconductor basic materials, such as Silicon, and metals compatible with current fabrication technologies. In this sense, considerable efforts have been made to obtain an efficient photodetector that can be easily integrated and processed through standard electronic manufacturing steps.

Basically, photodetector (PD) is a device that converts optical signals into electrical signals, thus it is also known as O/E convertor. The most used types of photodetector in optical communication systems are semiconductor-based photodetectors, usually called as photodiodes, because of their high detection efficiency, fast detection speed, and small size [13]. Photodiodes, like the structures of laser diodes, are based on the p-n junctions or p-i-n junction, where the diode is integrated with a wide, undoped intrinsic semiconductor region between a p-type semiconductor and an n-type semiconductor region. This intrinsic region enhances detection speed.

PD can be used in different configurations for different purposes:

- Single light sensors: A single sensor is used to detect overall light levels, it is useful to quantify the total optical power or light intensity;
- 1-D array light sensor: A line of PD is used to quantify the distribution of optical power or light intensity along a line. Combined with a wavelength splitter it can be used in a spectrophotometer; and
- 2-D array light sensor: A NxM matrix of photodetectors can be used to form images with NxM resolution.

**Figure 1:**
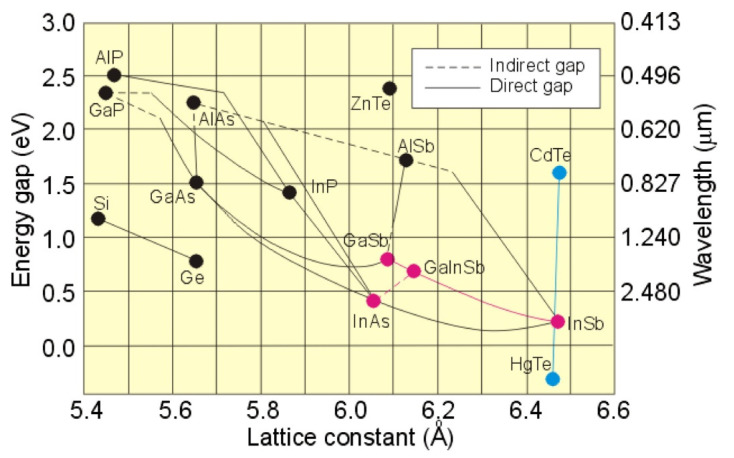
Composition and wavelength diagram of semiconductor material systems (Reprinted from [Rogalski, A.; Antoszewski, J.; Faraone, L. Third-generation infrared photodetector arrays. *J. Appl. Phys.* **2009**, *105*, 091101, doi:10.1063/1.3099572], with the permission of AIP Publishing).

Photodetectors are built by semiconductor materials which are able to absorb emitted photons and generate electron–hole pairs under the light excitation. A typical configuration is made of inorganic semiconductors [14], for example, GaN-based photodetectors are used for the ultraviolet sub-band (0.25–0.4 μm), Si-based photodetectors are implemented for the visible sub-band (0.45–0.8 μm), and InGaAs-based photodetectors are developed for near-infrared (NIR) sub-band (0.9–1.7 μm). An interesting and explicative graph that summarizes composition, energy gap and wavelength for most used material systems for infrared (IR) photodetectors is reported in Figure 1 [15], while some physical and optical properties at room temperature of narrow gap semiconductors are reported in Table 1 (Energy gap, E_g_, quantum efficiency, n_i_, dielectric permittivity, ε, and magnetic permeability, μ_e_ and μ_h_).

While the InSb has narrow band gaps in the mid-wave IR (MWIR), where it dominates as a semiconductor for PDs, PDs based on Hg_1-x_Cd_x_Te (MCT) have the great advantage of being able to tune the wavelength of operation from MWIR to long-wave IR (LWIR), adjusting the composition.

As it is known, semiconductor materials have a crystalline solid structure, but to have a device with optimal performance, this structure must not have defects and it should be single crystal. To achieve this, crystals must be grown by coupling the crystal lattice to that of the substrate. This is achieved either with molecular beam epitaxy (MBE) or chemical vapor deposition (CVD) and involves high costs for industrial production. Using heterostructured semiconductors, whose molecular structure has a broadband super-lattice, it was possible to obtain infrared absorption with intersubband transitions within one of the wells of the superlattice (GaAs/AlGaAs). PD based on this effect are called Quantum Well Photodetectors (QWP) [16] or Strained Layer Super-lattice Photodetectors (SLSP). Another approach has been the development of infrared quantum dot detectors (QDIP) based on self-assembled epitaxial quantum dots [17,18,19,20,21]. However, initially the use of QD in photodetectors presented two main difficulties: Limited control of QD size and low density of QD grown epitaxially. To date, InSb and MCT are the most used materials for the production of commercial quantum detectors [22]. Such quantum detectors exhibit high performance with high efficiency and high speed. However, the production of these detectors continues to be very expensive. In addition, considering their low operating temperature, they require a cooling system, so their use is limited to defense and astronomical research.

There is a plenty of materials studied in different configurations to realize an efficient photodetector, however most of them show complexity, high cost and difficulty to integrate with the standard CMOS process technologies. The research of alternative solutions that will address these problems requires a new active materials development or, in alternative, exploits the already known materials generally used for opto-electronic integration. Significant progress has been made in the past decade, overall using nanocrystal formations and colloidal quantum dot for IR sensing developments [23,24].

For these reasons, we focus our review paper on the current state of photodetectors based on group IV and colloidal semiconductors, which allow high compatibility with CMOS process and good performance.

## 2. Figure of Merits for Characterizing Photodetectors

In order to characterize the performance of photodetectors and compare different kinds of PD, some key figure-of-merit parameters are typically used. The main ones are summarized below [25,26]:

- *Quantum efficiency (QE)*: is the number of carriers (electrons or holes) generated per photon of a given energy. There are two types of QE: Internal quantum efficiency (IQE) that represents the number of charge carriers collected by the PD to the number of absorbed photons of a given energy, and external quantum efficiency (EQE) that is the number of charge carriers collected by the PD to the number of incident photons of a given energy.
- *Responsivity (R)*: The ratio of photogenerated current *I_ph_* to input light power *P_in_*, indicating the electrical response of an optical signal in units of A W^−1^,
  (1) $$R=\frac{I_{ph}}{P_{in}}$$
- *Spectral response*: Describes the responsivity of a PD as a function of photon frequency.
- Specific detectivity (D*): Represents the ability to detect weak optical signals. D* depends on the specific measurement conditions comprising the bias voltage, operating temperature, wavelength and modulation frequency. It is expressed in units of cm Hz^1/2^ W^−1^ (Jones),
  (2) $$D^{*}=\frac{R{\left(A\Delta f\right)}^{1/2}}{i_{n}}$$
  where *A* is the effective area of the device, Δ*f* is the electrical bandwidth and *i_n_* is the noise current that includes the shot noise from the dark current, Johnson noise and flicker noise. However, D* is generally considered in the shot noise limit, thus the Johnson noise and flicker noise can be neglected. Thus, Equation (2) can be simplified as $D^{*}=RA^{1/2}/{\left(2eI_{dark}\right)}^{1/2}$, where *e* is the electron charge, and *I_dark_* is the dark current.
- *Response time*: The time needed for a PD to rise or fall (*τ_r_*/*τ_f_*) from 10% to 90% or 90% to 10% of the final output.
- *Noise-equivalent power (NEP)*: Indicates the lowest amount of light power needed to generate a signal comparable to the noise of the device (i.e., a signal-to-noise ratio of ≈1) when the electrical bandwidth of the noise measurement is equal to 1 Hz. The NEP (W Hz^−1/2^) is proportional to the reciprocal of D*,
  (3) $$NEP=\frac{{\left(A\Delta f\right)}^{1/2}}{D^{*}}=\frac{i_{n}}{R}$$
*−3 dB bandwidth (f_-3dB_)*: Defined by the incident light modulation frequency at which the output signal is half-attenuated respect to its value under continuous wave illumination.
- *Linear dynamic range (LDR):* The range of incident light for which the detector responds linearly.
- *Fill factor:* The ratio of a light sensitive area of a pixel to its total area, typically it characterizes an image sensor array. The effective fill factor can be increased, often to nearly 100%, by using microlenses.

In an ideal photodiode, each photon of an input optical signal is immediately converted into a free electron and the generated current is linearly proportional to the input optical power. However, in real photodiodes, a lower absorption of photons and a leakage in carrier collection into the semiconductor material cause a non-perfect conversion in electrons of incoming photons. Moreover, the electric structures show an equivalent parasitic RC and the carrier transient effect limits the photodetection speed. Thus, the detection speed and the responsivity of a photodiode depend on the material quality, the bandgap structure of the semiconductor, the design of the device photonic and the electrodes [13].

Also, light reflection effect on the photodetector surface produces a leakage into the transduction, thus the PD, or the array of PD, is typically covered by an illumination window with an anti-reflection coating, reducing this effect. Furthermore, in an actual photodiode shot noise, thermal noise, and dark-current noise generates a signal-to-noise ratio (SNR) degradation in the photodetection process, which incisively affects the performance of an optical communication system. By increasing the load resistance, thermal noise can be decreased. Also, improvement of material quality and junction structure optimization can reduce the reverse saturation current and, then, dark-current noise. Nevertheless, shot noise is a white noise generated by the photo detection process itself and cannot be reduced. It is called the quantum limit of the optical system and it represents the fundamental limit of the photodiode performance.

Often, photodetectors are classified on the basis of the mechanism involved in the detection. As well know, the interaction between light and matter strongly depends by the photon energy and the band structure of the material constituent the photodetector. Then, effects of the light-matter interaction exploited for the photon-carrier transduction in PDs are summarized as following [25,26,27]:

- *Photoemission or photoelectric effect*: Energy of photons supplies exactly the energy gap from the conduction band to free electrons, increasing the mobility of electrons.
- *Thermal effect*: Energy of photons supplies to mid-gap transition states then an electron decay back to lower bands, generating phonon and thus heat.
- *Photochemical effect*: In some materials, photons can induce a chemical change as crosslinking or the destruction of a chemical bond.
- *Polarization effect*: In some materials, photons can cause changes in polarization states, which can change the refractive index or induces birefringence effects.
- *Weak interaction effects*: Photons can lead to secondary effects such as gas pressure changes or photon drag [28,29].

## 3. Group IV Semiconductors

**Figure 2:**
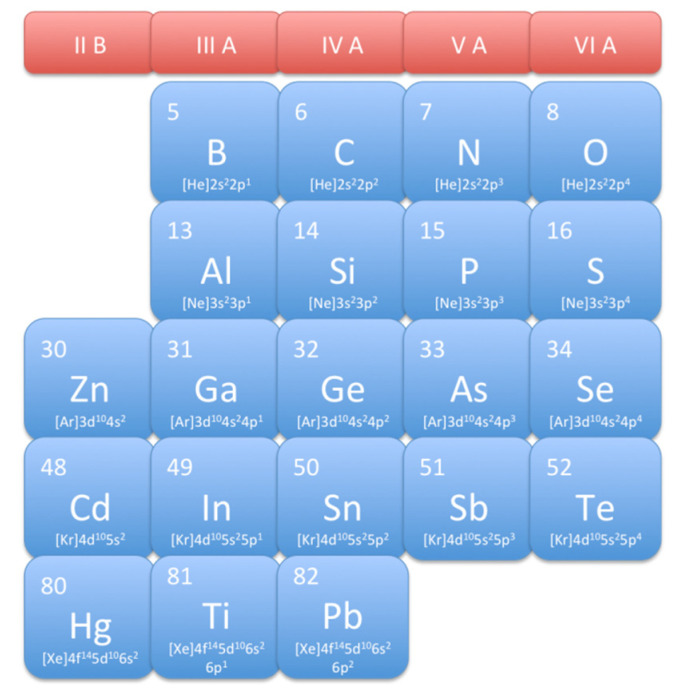
Table of elements involved in semiconductor based photodetectors (PDs) production.

The main semiconductors used to realize a photodetector, are summarized in the table of elements reported in Figure 2; in particular, the group IV semiconductors are currently the main materials for electronic and optoelectronic devices fabrication. Historically, efficient detection is provided by exploiting the direct bandgap of all III–V semiconductors; however, the integration of materials outer to group IV semiconductor with Si integrated circuit (IC) is complicated [30]. As example, in an integrated system the PD has to be closely connected to the biasing system and the electronic amplifying circuits [31], in order to exhibit the best possible performance.

The compatibility with Si technology of the IV Group semiconductors together with the efficient near-infrared light detection, enable fabrication of PDs and Si CMOS circuits in a simultaneously and monolithically integrated way [32]. Among PD systems, metal-semiconductor-metal (MSM) structures, with group IV semiconductor, are promising candidates for sensor optoelectronic integrated circuits (OEICs) due to the low detector capacitance, large device bandwidth, internal gain and, obviously, easiness of integration with electronic circuits. In spite of that, a lower bandgap increases the dark current associated with a low Schottky barrier in MSMs where the semiconductor is Ge or Si. As reported before, in order to limits the dark current effect, a reverse bias has to be applied leading to an extra power consumption. The connected heat generation increases the already high temperature of the Si IC substrate that absorbs the heat of the whole device. In particular, in Ref. [33] authors show both theoretically and experimentally a significant suppression of MSM-PD dark current by applying asymmetric metal electrodes and by modifying the Schottky barrier heights.

A similar technique has also been applied for MSM III-V PDs [34]. On the other hand, the only use of a wider bandgap semiconductor layer within the metal-semiconductor contacts can increase the Schottky barrier in MSMs and, thus, enhance the dark current effect [35].

Bulk silicon is naturally important for its main role in integrated circuits, but substantial challenges also derive from other elements by the group IV bulk materials and their alloys, nanocomposites, nanostructures, films of different thickness, and heterostructures. Advances in device performance are obtained by means of new defect engineering production, novel growth techniques, and improvements in diagnostic tools.

The advantage to consider group IV semiconductors is that they are widely studied thus modeling of defect generation, modeling of crystal growth, growth of group IV alloy crystals, low quality polycrystalline silicon refinement, including control of dopants and wafering technologies, and defect evolution in wafering processes are all well-known issues. Moreover, nanostructures of/on group IV semiconductors are largely developed, as well as modeling and simulation of epitaxial structures, heterogeneous integration of Si or Ge with III-V epitaxial device quality layers, growth of 2D materials (e.g., graphene, silicene, and germanene) on silicon, deposition of amorphous and crystalline thin layers and silicon membranes. Fundamental research on point defects and extended defects in group IV semiconductors is continuously in progress, as well as the study of dislocation engineering by substrate and process optimization. Additionally, considering the several technological applications for group IV semiconductors, such as silicon on insulator (SOI) devices, high speed and high frequency electronic devices, photonics and light emitting devices, and power devices, it is natural to invest resources to enhance the performance of devices based on these semiconductors.

### 3.1. Si

Although in the visible spectrum the PDs in Si have reached the phase of commercial development, in NIR band the use of Si for the realization of PD is difficult due to its transparency at wavelengths greater than 1.1 μm. Generally, the integration of germanium (Ge) in a SiGe heterostructure is used to overcome this drawback [36,37], however a reticular superlattice mismatch of 4.3% leads to a very high leakage current, affecting the performance of the device. A two-step epitaxial growth technique can mitigate these effects [36,37] but, unfortunately, does not completely remove the defect center responsible for the high leakage current. Furthermore, the grown buffer layer gives rise to thermal and flatness problems [38], which prevent the possibility of a monolithic integration of Ge on Si. Additionally, in p-i-n structures the Ge needs a very thick intrinsic layer (especially with respect to the gallium arsenide (InGaAs)) because at 1550 nm the Ge shows a lower absorption.

In this paragraph, we present a brief overview on all-Si photodetectors at NIR wavelengths. In these PDs, the most used physical effects, that allow the absorption at sub-band wavelength, are the internal photoemission absorption (IPA) in presence of positive charged particles or molecules, two photon absorption (TPA), mid-bandgap absorption (MBA), and surface state absorption (SSA). A quantitative comparison of PDs in the literature, divided by absorption effects are shown in Table 2.

To improve the Si based photodetectors performance, many approaches have been considered and proposed. Among them, encouraging results have been obtained by exploiting the internal photo absorption effect (IPA), that is, the use of photon-assisted transmission of hot carriers across a potential barrier at metal-semiconductor interfaces. Several new structures have been reported in literature combining, for example, IPA with nanoscale metallic structures, comprising Si nanoparticles (NPs) [39], metal stripes allowing surface plasmon polaritons (SPPs) [40,41], metallic gratings [42] and new structures based on two-dimensional materials (like graphene) capable to substitute metal in the Schottky junction [43]. Moreover, as reported in some complete review on this topic [44,45], due to the unipolar nature of the Schottky junction, IPE based PDs are very fast and can be monolithically integrated with Si-based CCD for IR applications [46].

A 50 Gb/s pure silicon waveguide photodetector at 1310 nm datacom wavelength has been also developed [47]. Its working principle is based on a combination of TPA, SSA, and photon assisted tunneling (PAT) effects in a Si PN junction; responsivity was found to be 0.6 A/W, dark current was of 850 nA at 5.84 V reverse bias and eye SNR was of 7.3.

Nanostructured photodetectors have attracted a lot of interests in the recent past, due to their large surface-to-volume ratios and the reduced dimension, that lead to superior performances in terms of responsivity and photoresponse gain with respect to bulk crystals. Indeed, the large surface areas of nanostructures gives rise to a prolonged photocarrier lifetime leading to higher sensitivity and responsivity. Moreover, nanostructured photodetectors show a fast response speed owing the low dimensionality which shortens the transit time during photoelectric processes [48,49,50], and good compatibility with semiconductor technology. On this line of research, 1D inorganic nano-wires (NWs) PD have demonstrated high responsivity, fast response, specific detectivity, high spectral selection, good flexibility, and low energy consumption [51]. The first avalanche photodetectors (APD)-based nanoscale p–i–n junction Si nanowire (SiNW) was demonstrated in 2006 and this device showed an avalanche breakdown mechanism with large reverse bias [52]. After that, several studies were performed on Schottky junction NIR photodiodes and nano-heterojunction NIR-PDs based on SiNW [53] combined with metal films (e.g., Cu [54], Ag [55] and Au/Cr [56]), graphene oxide [57], and other nanostructured semiconductor comprising carbon quantum dots [58].

With the aim to improve the excitation efficiency, Yatsui et al. [59] investigated optical near-field excitation that is confined in a nano-scale. Here, due to the uncertainty principle, the interband transitions between different wave numbers are excited; therefore, optical near-field can directly excite the carrier in the Si indirect bandgap. The authors developed a lateral Si p–n junction with Au nanoparticles as sources to generate the field confinement and they demonstrated that the photo-sensitivity rate increases of 47.0%. Moreover, the far-field excitation is eliminated when the thin lateral p–n junction is used, confirming a 42.3% increase in the photosensitivity rate [59].

Even though the Si-based PD limitation can be partially solved by femtosecond laser processing, the surface defects and carrier activation rates produces a large dark current and narrow spectral response, which are drawbacks. To overcome this, Huang et al. [60] have recently proposed a rapid thermal annealing and hydrogenated surface passivation demonstrating, at optimal conditions, a sub-bandgap responsivity of 0.80 A W^−1^ for 1550 nm at 20 V at room temperature; these results are comparable with commercial Ge based PDs and higher than previously reported Si photodiodes. This Si-based photodetector shows a spectral range of 400 ÷ 1600 nm and a competitive detectivity (1.22 × 10^14^ Jones at −5 V). Finally, its responsivity was 1097.60 A W^−1^ for 1080 nm at 20 V, which is the highest reported to date in Si photodetectors, thus opening good prospects for black silicon in IR light detection, night vision imaging, and fiber-optic communication [60].

### 3.2. Ge

Germanium has a direct bandgap energy of 0.8 eV, high optical absorption over the 1.3–1.55 μm wavelength range used for fiber-optic communications [61] and a perfect compatibility with the conventional silicon processing technology. For these characteristics, Ge-based photodetectors have been widely studied since the end of 1990s [62,63,64,65] and, currently, Ge PDs performance are comparable to that of III-V materials.

For nanophotonics applications, Ge photodetectors are often integrated at the end of optical waveguides, indeed in this configurations light absorption takes place along the optical mode propagation direction and perpendicularly to the carrier collection path. Moreover, both Ge homo-junction [62,63,64] and Si-Ge-Si hetero-junction [65,66,67] photodetector architectures, which are made with a p-i-n junction where light absorption occurs in the intrinsic regions, have been experimentally demonstrated. A lateral hetero-structured silicon-Ge-silicon (Si-Ge-Si) junction was realized to obtain a p-i-n waveguide photodetectors working under low reverse bias at 1.55 μm [67]. Such hetero-structured Si-Ge-Si photodetector allows a superior signal detection of high-speed data traffic, having efficiency-bandwidth products of ∼9 GHz at −1 V and ∼30 GHz at −3 V, with a leakage dark current as low as ∼150 nA. For conventional 10 Gbps, 20 Gbps, and 25 Gbps data rates, a bit-error rate of 10^−9^ has been obtained, leading to optical power sensitivities of −13.85 dBm, −12.70 dBm, and −11.25 dBm, respectively. An interesting solution for the Ge-on-Si PD was proposed by Tzu et al. [68]; the authors developed arrayed germanium-on-silicon waveguide photodetectors for high-power analog applications. Arrays of photodetector with 2, 4, and 8 elements allow to have output powers of −0.4 dBm, 10 dBm, and 14.3 dBm at 18 GHz, 12 GHz, and 5 GHz, respectively. The 4-photodiode array shows a 5-dB improvement over a single PD. An integrated 20 GHz receiver based on a couple of balanced PDs and a Mach-Zehnder delay line interferometer has been also demonstrated in an optical phase-modulated link.

**Figure 3:**
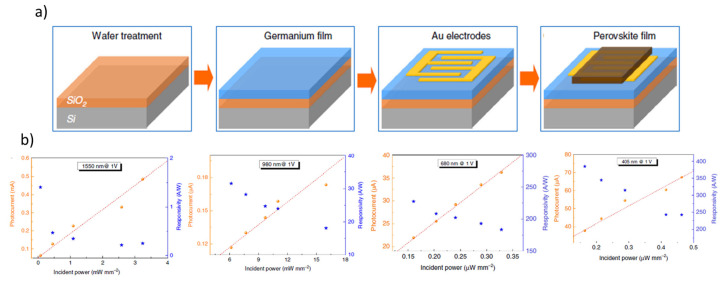
(**a**) Schematic illustration of the germanium/perovskite heterojunction device fabrication process. From left to right: a cleaned SiO_2_/Si substrate, growth of a germanium layer by molecular beam epitaxy (MBE), Au electrode deposition on the substrate, perovskite layer construction by the vapor-solution method. (**b**) I_ph_ and R values under an illumination wavelength of 1550 nm, 980 nm, 680 nm and 405 nm, from left to right, respectively (adapted from [69]).

Recently, several solution-processed has been proposed in order to obtain good performance of PDs based on Ge. For example, Hu et al. successfully fabricated a perovskite/germanium heterojunction photodetector with excellent photo-response properties [69]. The heterostructure device was realized by a uniform and pinhole-free perovskite film deposited on top of a single-crystal germanium layer, as illustrated in Figure 3a. Results demonstrated a broad spectrum compared with the single-material-based device, as showed in Figure 3b where the photon response properties are characterized from the visible to near-infrared spectrum. In particular, at optical fiber communication wavelength of 1550 nm this device presents the highest responsivity of 1.4 A/W, while at a visible light wavelength of 680 nm, it exhibits considerable detectivity and responsivity of 1.6 × 10^10^ Jones and 228 A/W, respectively. The authors have been also estimated the photoconductive gain of the realized device. The gain is defined as [69]

(4) $$G=\frac{I_{ph}}{I_{PI}}=\frac{\tau }{t_{p}}=\frac{\tau \xi \left(\mu_{e}+\mu_{h}\right)}{L}$$

where *I_PI_* is the primary photocurrent, *τ* is the carrier lifetime, *t_p_* is the carrier transit time through the electrodes, *ξ* is the applied electric field, *L* is the width of the channel, *μ_h_* and *μ_e_* are the hole and electron mobility, respectively. Some of the photogenerated electrons are moved from the perovskite layer to the germanium layer when visible light excitation are considered. Respect to the perovskite, the long carrier lifetime (≈200 μs) and ultra-high electron mobility (≈3800 cm^2^ V^−1^ s^−1^) in the germanium layer lead to an enhanced gain of about 10^4^ in this heterojunction photodetector.

In 2018, a PDs based on Ge QDs fabricated on Ge substrates has been demonstrated with a broadband photoresponse spectral range (λ = 400–1550 nm) [70]. Its responsivity at room temperature was up to 1.12 A/W, the obtained internal quantum efficiency was IQE = 313%, higher than conventional Si and Ge photodiodes, specific detectivity at room-temperature was D* ≈ 210^10^ cm Hz^1/2^ W^−1^ both at visible λ = 640 nm and telecom λ = 1550 nm wavelengths. When the operating temperature and incident power were decreased, sharply improved performance were obtained, indeed at T = 100K for an incident power of 10 nW at λ = 1550 nm, the device showed D* = 1.110^12^ cm Hz^1/2^ W^−1^ and IQE = 1000% [70].

In a very recent work, MSM NIR photodiodes are fabricated on the epitaxial uniform Ge film with a grain size of up to 12 μm, with two diverse electrodes and two different surface passivation interlayers [71]. When electrodes were made of amorphous-Ge (30 nm)/Al (100 nm) and TiO_2_ (5 nm)/Au (80 nm), MSM devices show an average spectral responsivity of 0.50 ± 0.16 A/Wand 0.35 ± 0.09 A/W at 1550 nm and voltage bias of −3V, respectively. The maximum spectral responsivity reported was of 0.78 A/W and 0.48 A/W, respectively.

Considering the recent encouraging reported studies and results, together with a well-tested compatibility with the conventional CMOS technology, Ge can be considered a material on which to continue investigating.

### 3.3. Carbon

The carbon family comprises fullerene [72], carbon nanodots (CQDs) [73], carbon nanotubes (CNTs) [74,75], graphene [76], and graphene quantum dots (GQDs) [77]. Considering the superior and uniquely chemical, optical, physical, mechanical, and electronic properties of these innovative materials [78], they have attracted a growing research interest in the last couple of decades.

In particular, CNTs and graphene have shown unique potential for a broad range of photodetectors from the ultraviolet to the THz [79]. In this section we introduce the basic properties of both CNTs and graphene based photodetectors that have been realized in the last few years. The performance status of these detectors is reported, and their potential for further improvement is discussed.

#### 3.3.1. Carbon Nanotube

CNTs are tubes made of carbon with diameters in the order of nanometers. When diameters are in the range of a nanometer, carbon nanotubes are called single-wall carbon nanotubes (SWCNTs). Respect to devices made with other semiconductors materials, CNT based photodetectors show a fine IR detection together with low fabrication cost, ease of production and scalability, actually making CNTs highly promising nanomaterials for multiwavelength, room-temperature IR detection applications [80]. Up to now, several IR photodetectors based on both individual CNT and CNTs films [81,82] have been successfully proven. Moreover, few years ago, a prototype of infrared camera was realized based on single CNT photodetectors [83].

In order to develop an efficient CNT photodiodes, based on photovoltaic mechanism, an efficient separation electron–hole pairs into free carriers should be obtained as well as an increase of the absorption cross-section of the device. In particular, the first studies on the realization of CNT photodiodes demonstrated that electron–hole pairs separation and photocurrent generation can be obtained by the built-in field in a CNT Schottky and p-n diode [84,85,86]. Moreover, even if CNT photodiode attracts a lot of research interest to study the mechanisms of photocurrent generation along with many other optoelectronic properties [86], the extremely small absorption cross-section of individual CNT devices leads to a low sensitivity [87]. Nevertheless, significant improvements have been reached in the recent past, allowing to increase the responsivity from ~pA/W to ~A/W [85,88].

Since the pioneering work proposed by Lee et al. in 2005 [85], where the first realization of individual semiconducting SWCNT p-i-n diode was demonstrated, several other studies were performed on these kind of devices. In detail, the device realized by Lee et al. consisted in a SWCNT where two split gates where formed in adjacent parts by using electrostatic doping to realize n-region and p region, respectively. The photocurrent generated under IR illumination with a power density ~20 W/cm^2^ was of ~5 pA, whereas the fill factor (FF) and the efficiency η were of 0.33–0.52 and ~0.2%, respectively.

In 2011 a photodetector based on SWCNT/C(60) in heterojunction photovoltaic was demonstrated with an EQE of 12% and a detectivity approaching ~10^12^ cm Hz^1/2^ giving a proposal for the development of the next-generation high-performance solar cells based on CNTs [89]. Thin films of highly purified semiconducting CNTs were used as NIR optical absorbers in heterojunction photodetector and photovoltaic devices with the electron acceptor C(60), leading to a 10-fold enhanced gain in zero-bias quantum efficiency and significant gains in power conversion efficiency respect to the implementations of more electrically heterogeneous CNT/C(60) devices. In particular, the exciton migration along an effective length scale in the nanotube films has been related to the device efficiency, validating the high IQE through photoluminescence quenching, and demonstrating that for diameters <1.0 nm the driving force for exciton dissociation at the fullerene-fullerene heterointerface is optimized [89].

Liang et al. demonstrated a ∼6-fold higher optical absorption in a single-tube diode photodetector monolithically integrated with a Fabry–Pérot microcavity, due to the confined effect of the designed optical mode [90]. The obtained photodetector shows a spectral FWHM of about 33 nm at a signal wavelength of 1200 nm and a higher suppression ratio until a power density of 0.07 W cm^–2^ and photocurrent of 5 pA. Moreover, taking advantage by the theory of the “resonance and off-resonance” cavity, the authors realized cavity-integrated chirality-sorted CNT-film detectors for specific target signal detection, operating at zero bias and resonance-allowed mode. By integrating multiple array detectors on a chip working at different target signals, a multiwavelength signal detector system can be obtained; this device can be used in the fields of colour imaging, monitoring, signal capture, biosensing, and on-chip or space information transfers [90].

**Figure 4:**
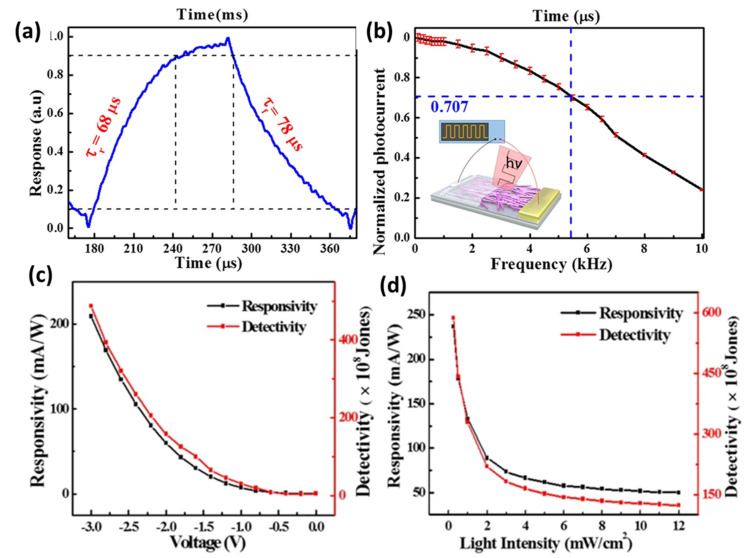
Photoresponse of the PD. (**a**) Single normalized cycle measured at 5 kHz for evaluating both rise (τ_r_) and fall time (τ_f_). (**b**) Normalized photocurrent versus switching frequency. (**c**) Responsivity and detectivity of the PD as a function of bias voltage. (**d**) Responsivity and detectivity of the PD as a function of light intensity. (Adapted from [80]).

A broadband nano-photodetector based on single-layer graphene-CNT thin film (SLG-CNTF) Schottky junction was studied and developed by Zhang et al. [80]. SLG-CNTF device shows peak sensitivity at 600 and 920 nm, a fast response speed (see Figure 4a; τ_r_ = 68 μs, τ_f_ = 78 μs, much faster than several seconds response time of the graphite quantum dots/graphene heterojunction [91]) and good reproducibility for switching frequencies in the range 50–5400 Hz (the normalized photocurrent at different frequencies shown in Figure 4b). The responsivity and detectivity at different bias voltages were investigated, as shown in Figure 4c, where is evident that both parameters increase with decreasing bias voltage. Moreover, the authors studied the photocurrent at different light intensities (from 0.25 to 12 mW cm^−2^) finding a high dependence of the photocurrent of the SLG-CNTF Schottky junction photodetector on the intensity of incident IR. The trend of responsivity and detectivity as a function of light intensities is reported in Figure 4d where a saturation of both parameters is evident at high intensity because the carrier recombination rate is reduced at high light intensity [92].

Recently, CNT/Si photodetectors in two electrode configurations for photovoltaic and photoconductive operations were studied under nanosecond light pulse [93]. A linear dependence of the photocurrent as a function of the light pulse energy was observed in photovoltaic mode with rise time of 20 ns, while when the device operates in photoconductive mode, the maximum photocurrent increases up to 30 times with a gain in the number of photogenerated charges till 200% and a reduction in the time response below 10 ns. At carbon nanotube/Si interface a Schottky junctions is formed, leading to a fast response, as pointed out by the current-voltage characteristics measured as a function of the temperature [93]. These findings open the possibility of CNT/Si photodetectors used as avalanche photomultipliers.

#### 3.3.2. Graphene

Since its discovery in 2004, graphene, a single layer of carbon atoms arranged in a closely packed two-dimensional honeycomb lattice, has attracted a lot of research interests due to its intriguing physical properties such as ultrahigh mobility (200,000 cm^−2^/V∙s at room temperature), high electrical conductivity (10^8^ S/m) and an exceptionally large thermal conductivity up to 5300 W⋅m^−1^⋅K^−1^ [94]. Moreover, graphene has would be involved in fabricating thinner and faster response speed optoelectronic devices [95,96]. Conversely, even if graphene can act as an absorber in ultrafast and ultra-broadband detectors, graphene-based photodetectors show relatively low responsivity, since a single sheet of carbon atoms has a relatively low absorbance (only 2.3%) from the ultraviolet (UV) to near infrared (NIR) region and short light-matter interaction length. Moreover, in pure graphene, due to its gapless nature, lifetime of excitons is extremely short giving rise to fast carrier recombination, which limits the efficient generation of photocurrent or photovoltage [97,98].

To overcome this problem, Schottky junction based photodiodes have been proposed in several combination of nanostructures such as II-VI (ZnO [99,100], ZnTe [101]), IV (Si [102,103], Ge [104], GeTe [105]), and III-VI (GaN [106], InAs [107], Al_2_O_3_ [108]), leading to improvements of both sensitivity and response speed. A very fascinating feature of the Schottky junction is that, due to its photovoltaic characteristics, most of the semiconductor-graphene devices are able to detect light irradiation without power supply [80]. An interesting and complete review of PDs based on graphene/semiconductor hybrid heterostructures, comprising device physics, design, performance, and process technologies for the optimization of PDs was recently published by Shin and Choi [109]. Considering that generally an all-Si approach is desired to take advantage by the existing CMOS technologies, a detailed review on the emerging field of the NIR internal photoemission effect-based graphene/Si PDs is presented by Casalino [110].

Regarding silicon-graphene heterostructures, in 2018, Periyanagounder et al. [111] realized and characterized a graphene/silicon (Gr/Si) (2D/3D) van der Waals heterostructure for high-performance PDs. Here, graphene works as an efficient carrier collector and Si as a photon absorption layer, allowing to obtain a barrier height of 0.76 eV and good performance as a self-powered detector under the mechanism of photovoltaic effect, working at 532 nm with zero bias. The authors measured a responsivity up to 510 mA W^−1^, a photo switching ratio of 10^5^ and a response time of 130 μs. These good results have been ascribed to the Schottky barrier which extends the lifetime of photo-excited carriers leading to a fast separation and transport of photoexcited carriers [111].

**Figure 5:**
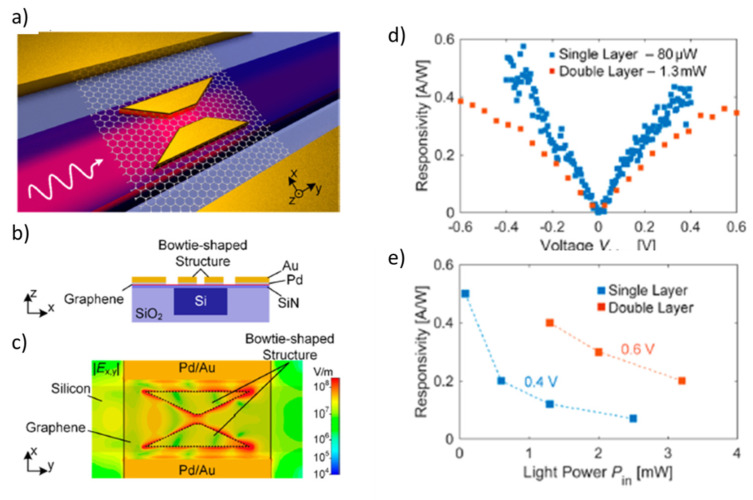
Plasmonically enhanced waveguide-integrated graphene PD. (**a**) 3D perspective view of the PD. (**b**) Cross sectional view of the graphene PD. (**c**) In-plane view of the magnitude of Ex and Ey electric fields, obtained by 3D full-wave finite-element method (FEM) simulations. (**d**) Responsivity of the PD as a function of bias voltage for a single-layer (blue scatters) and a double-layer graphene device (red scatters). (**e**) Responsivity of the PD as a function of light input power. (Adapted from [112]).

More recently, a compact PD operating as a metal–graphene–metal photoconductive detector has been co-integrated with a Si photonic waveguide, demonstrating extremely efficient and high-speed plasmonically enhanced waveguide-integrated photodetector [112]. A Si waveguide allows the light enters and evanescently coupled into the graphene-based PD, made with a 6 μm long layer of graphene. Then, the in-plane electric field is enhanced by the integrated nanosized bowtie-shaped metallic structures, as reported in Figure 5a,b. Simulation of magnitude of x and y components of the electric field was performed, and results are shown in Figure 5c, where is clearly visible a strong plasmonic field enhancement along the edges and in the gap of the bowtie-shaped structures. The single-layer graphene device exhibits a high external responsivity of 0.5 A/W under a bias of −0.4 V as measured with an input power of 80 μW, while if double-layer graphene device is considered, a higher input power can be reached obtaining 0.4 A/W at a bias of −0.6 V for an input signal of 1.3 mW (see Figure 5d,e). Moreover, the device demonstrates a fast photoresponse up to at least 110 GHz [112].

The results obtained so far, together with the growing study of high performance self-powered silicon-graphene heterostructures detectors paves the way for the technological implementation of Si-based monolithic optoelectronic devices for highest speed communication applications.

## 4. Colloidal Semiconductor

As mentioned before, materials nanostructuring offers the possibility to enhance material properties and devices performances. In the case of PDs, bottom up and top down fabrication of nanocrystals (NCs), nanoparticles (NPs), nanowires (NWs) etc. have been largely experienced to improve resistivity, work function (WF), bandgap or dielectric constant (ε). At the same time, dopant percentage, nanocomposition and several kind of deposition methods have been exploited to realize PD with low dark current, high EQE and wide band absorption.

### 4.1. Metal Oxide

Colloidal metal oxides have attracted much attention as based semiconductor material, because of their high stability [24,113,114]. Moreover, colloidal metal oxides showed adjustable electrical properties as resistivity, work function (WF) and dielectric constant (ε), which can be tuned by controlling vacancy density, doping material or other controllable parameters [115]. Finally, their showed tunable optical properties (e.g., photoluminescence, absorption, and transparency) tuning the band gap by changing alloys and engineering of components [116,117].

In PDs, colloidal metal oxides are largely employed because of their wide band gap, large exciton binding energy, combined with the simplicity of fabrication on a large-scale, with low cost, and tunable physical and chemical properties [118,119,120,121].

**Figure 6:**
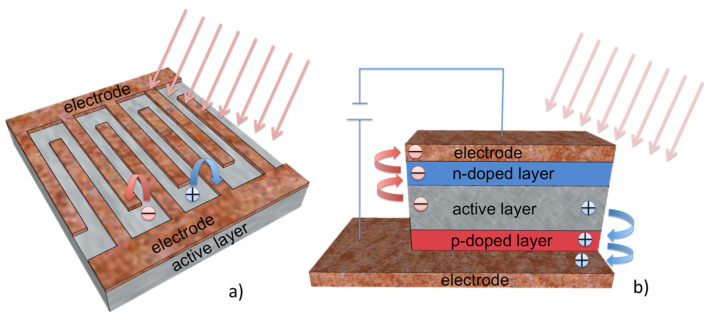
(**a**) Planar configuration and its basic working operation; (**b**) vertical configuration and its basic working operation.

Also in this case, PDs can be divided into planar PDs and vertical PDs, according to device design [122,123]. PDs in planar configuration consist of an electrode and one only active layers (MOs), as shown in Figure 6a. Under no illumination, planar PDs have small Schottky barriers at the contacts, while when they are illuminated, photons are absorbed and electron–hole pairs are created, resulting in a photocurrent, by applying a voltage. The applied voltage reduce the carrier transit time and the photocurrent increases (Figure 6a) [124]. In vertical PDs, a n-doped semiconductor layer and a p-doped semiconductor layer are added, as shown in Figure 6b. Under illumination, the doped layers transport electrons and holes, decreasing the carrier transit time and, thus, increasing the efficiency of the device (Figure 6b) [125].

Focusing on the active layer in planar PDs, in recent years, a lot of efforts have been employed to syntetize colloidal wide band gap MOs such as TiO_2_ [126] and ZnO [127], used in UV PDs [128,129]. Nowadays, colloidal nanowires (NW) and nanobelts (NB) offer excellent solutions for optoelectronic applications and in particular for PD.

In [130], the authors synthesize SnO_2_ NWs with a quite uniform diameter of about 26 nm, assemble a film by dip coating and fabricate a high performance PD device. In fact, it has excellent selectivity and light stability. Moreover, in [130] the EQE has been calculated with the follow equation:

(5) $$EQE=\frac{t_{life}}{t_{tran}}=\frac{hc}{e\lambda }\cdot \frac{\Delta I}{PS}$$

where *t_life_* and *t_tran_* are the lifetime of carriers and the transit time between electrodes, respectively, *h* is he Planck’s constant, *c* is the velocity of light, *e* is the electronic charge, λ is the incident wavelength, Δ*I* is the difference between the current and dark current, *P* is the light power density and *S* is the irradiated area of a single nanowire. For the present device, irradiated by 320 nm light at 0.91 mW cm^−2^, EQE has been calculated and a value of 1.32 × 10^7^ was obtained, four orders of magnitude larger than that of standard SnO_2_ photodetectors. This detector is proposed not only for optoelectronic applications, but also in the visible and UV spectrum.

The PD assembled in a film of In_2_Ge_2_O_7_ nanobelt with high quality monocrystals are expected to find wide optoelectronic applications in switches, optical sensors and generally in communication systems. In [131], the developed detectors showed high optical and electrical performance, i.e., EQE of 2.0 × 10^8^%, decay times of ~3 ms and responsivity of 3.9 × 10^5^ A/W, in addiction to high stability, reproducibility, sensitivity and selectivity.

PD based on NB of Nb_2_O_5_ showed high photosensitivity, selectivity to light and excellent photocurrent stability for >2500 s. The responsivity and EQE are determined to reach 15.2 A/W and 6070% respectively, demonstrating the potential of NB Nb_2_O_5_ in next-generation photosensors and PDs into the UV spectrum [132].

Zn_2_SnO_4_ (ZTO) has aroused great interest as a base material for PDs for its photodetection performance. In particular, ZTO is used as a PD in association with various polymers that are used to decorate the surface of ZTO, improving its ZTO properties.

In [133], it has been shown that by combining ZTO’s NWs with polydimethylsiloxane (PDMS) it is possible to exploit the transparency and flexibility of PDMS with its NWs photodetection functionality. Furthermore, the photoactive materials are chemically bound in the PDMS matrix and less exposed to air and therefore to oxidation. Compared to the NWs completely immersed in the polymer matrix, the decorated NWs have a significant improvement in the PD in terms of speed of response (less than 0.8) with a recovery time of around 3 s.

**Figure 7:**
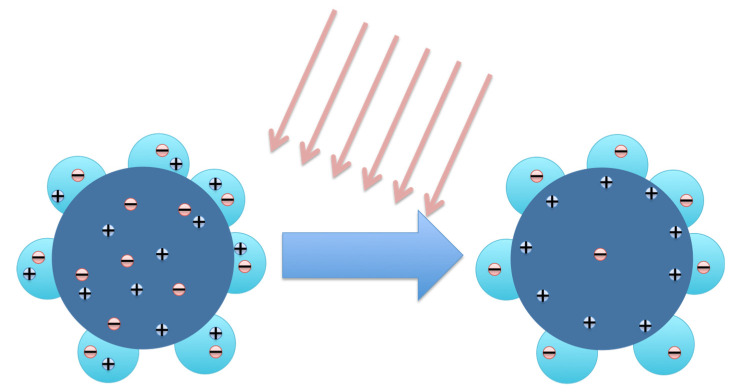
Sketch of carrier separation in the cross section of ZTO nanowires (NWs) decorated with ZnO quantum dots (QDs) upon illumination.

Another type of decoration of NWs has been presented by Li et al. in [134]. The authors manufactured a PD with heterojunction structure with high performance in the UV spectrum, in which the active material is made up of ZTO NWs decorated with quantum dots (QDs) in ZnO. the NWs decorated with ZnO are summarized in two steps. In Figure 7, a sketch of carriers separation in ZnO QD decorated ZTO NW PD on lighting is shown.

Compared to simple ZTO NWs, decorated QD NWs has 10 times higher photocurrent and response speed, with a light-dark current ratio up to 6.8 × 10^4^ and a photoconductive gain up to 1.1 × 10^7^, in addition to excellent stability.

Decoration of ZnO nanoparticles (NPs) has been also used to broader the absorption band and improve the photosensitivity. As example, in ZnO NPs the excess of Zn^2+^ ions toghedar with oxygen lack cause a great green photoluminescence [135,136], but coating the ZnO NPs with polyvinyl-alcohol, as done in [137], the ratio of photocurrent on dark current ranges from 3.8 × 10^6^ to 1.34 × 10^8^. In [138] simply changing the solution method of ZnO NPs into a coating spray, the authors shows that it is possible to tune the percentage of vacuum and zinc interstitial spaces. In this way, a significant broadening in visible light photo-detection is reached. Finally, in [139], Gogurla et al. synthesized gold decorated ZnO NPs colloidal spin coating solution. The fabricated film shows strong plasmon assisted SSA and scattering, leading to an increase of photoresponse by 80 times over the control simple ZnO NPs.

In the end of this paragraph, we would underline how colloidal NPs can be also easly integrated in a Si based heterojunction by drop casting; as done in [140], where authors synthesized In_2_O_3_ NPs and casted them on an n-type single-crystal Si wafer, showed an high photoresponse in visible and near-infrared regions. Infact in [140], in order to fabricate a heterojunction photodetector, thin films In_2_O_3_ NPs is deposited on a (100)-oriented 10 Ω cm n-type silicon by drop-casting and dried at 80 °C for 15 min under vacuum with multiple layers deposition and condensation steps. The final thickness of the In_2_O_3_ layer was 150 nm. Aluminum electrodes have been fabricated by means of standard lithographic process to make the ohmic contacts on the In_2_O_3_ nanoparticles layer and the back side of the Si wafer

### 4.2. HgTe and HgSe Nanocristals

In the infrared region, Hg nanocomposites have been widely explored to produce active materials in both planar and vertical PD. In particular, HgTe colloidal nanocrystals have been investigated in PD at telecom wavelength [141,142,143] and for solar applications [144,145] for their photoconduction properties [146].

In the NIR, typical applications are in communications technologies, biological imaging, and night imaging. Among possible colloidal nanomaterials to be used in NIR, HgTe NCs have reached enough maturity. In those materials, interband transitions assure IR absorption, see Figure 8 from [23].

For wavelengths up to 1.7 μm, performances of InGaAs based PD are again higher. However, Hg based nanocomposites are promising as extending the range of wavelengths and reducing cost [147]. In fact, if in PD based on InGaAs is possible to achieve extremely low dark current at room temperature (<20 fA), in colloidal NCs based devices currently reported, such dark current are not so low. However, colloidal NCs based PDs have typically faster detection times, then they are usually employed to flame detection [148], night imaging [149] and biological imaging [150] for which detection is mandatory sub-ms sampling times.

In this case, the using of decorated NPs, i.e., coupling the absorption of the HgX NCs with plasmonic structures, show improvements in detectivity, as done in [151,152,153]. In particular, coupling HgTe with gold nanorods, the absorption is increased respect to thin layers of colloidal NCs, and a three time detectivity is reported respect the undecorated ones.

However, the use of colloidal NCs has some challenges that need to be addressed to bring the IR NCs based PDs to industrial production. Main challenges are about materials properties, as well as the toxicity and the trial of large synthesis (>10 g). Moreover, the not well known band structure of new NCs implies difficulties to design device with optimized ohmic contacts and generally band alignments; the HgX NCs experience rapid oxidation in air, then either the NCs surface have to be chemically stabilized in air, or the material have to be processed in glow box and then encapsulated into a protective layer.

## 5. Conclusions and Outlook

Photodetectors can be used in a wide range of applications, such as medical imaging, nuclear safety guards, aerospace, high energy physics experiments, photonics, and astrophysics. For this reason, there is a great interest in developing high performance, cheap and compact light sensor devices.

In this article, we firstly reviewed recent research progress in the field of photodetectors based on Group IV semiconductors. Historically, Group IV semiconductors are the most used materials for electronics devices; this is especially true for silicon, which is considered the main material for high integration CMOS technology. For this reason, we have investigated the developments of photodetectors based on these semiconductors and presented in recent years. Several narrow band-gap elementary semiconductors (e.g., Si, Ge, graphene, and CNTs) have been considered and different solutions proposed in the recent literature are reviewed in order to obtain photodetectors with good performance. Overall, MBA-based waveguide photodetectors are showing better performance than devices based on other absorption mechanisms. The recent reduction in the size ability of NIR all-Si photodetector production has open the road to new structures such as SSA and MBA-based ring resonator PDs or TPA-based PhC nanocavity PDs.

Then, an overview on colloidal semiconductor based photodetectors has been also presented; as these materials represents the frontier in future research, promising new opportunities for high performance and high-density chip-scale photonic integration from UV to infrared spectral regions.

The two semiconductor groups taken into consideration represent one the story that can still evolve and bring new improvements and the other the central topic on which to invest in the future, respectively. However, additional studies are required to further improve the device performance.

We strongly believe that high performance photodetectors based on group IV or colloidal semiconductors monolithically integrated on Si showing better functionality and high integration density, may be proven in the next future.

## Figures and Tables

**Figure 8:**
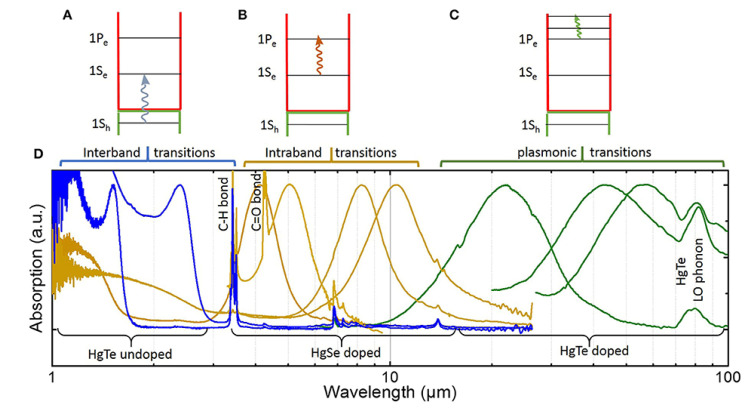
(**A**–**C**) Scheme for interband, intraband, and plasmonic transitions, respectively, in HgX NCs. (**D**) Absorption spectra HgX NCs (reprinted from [23]).

**Table 1:** Some physical and optical properties at room temperature of narrow gap semiconductors [15].

Material	E_g_ (eV)	n_i_ (cm^−3^)	ε	μ_e_ (10^4^ cm^2^/Vs)	μ_h_ (10^4^ cm^2^/Vs)
InAs	0.359	9.3 × 10^14^	14.5	3	0.02
InSb	0.18	1.9 × 10^16^	17.9	8	0.08
PbS	0.42	1.0 × 10^15^	172	0.05	0.06
PbSe	0.28	2.0 × 10^16^	227	0.10	0.10
PbTe	0.31	1.5 × 10^16^	428	0.17	0.08
In_0.53_Ga_0.47_As	0.75	5.4 × 10^11^	14.6	1.38	0.05
Pb_0.44_Sn_0.56_Te	0.1	2.0 × 10^16^	400	0.12	0.08
Hg_1−x_Cd_x_Te	0.07–0.25	(0.23–2.3) × 10^16^	16.7–18.0	0.6–1.0	0.01

**Table 2:** Some physical and optical properties at room temperature of narrow gap semiconductors.

Responsivity (A/W)	V_bias_ (V)	Operating Wavelength (nm)	Dark Current/Leakage	Effect	Ref.
4.6 × 10^−3^	−1	1550	3 nA	IPA NiSi_2_/p-Si Schottky barrier	[39]
8 × 10^−^^3^	−1	1550	∼3 nA	MBA Proton implantation	[40]
0.8 × 10^−3^	−0.1	1550	6 μA	IPA Surface plasmon polariton	[41]
64 × 10^−3^	−20	1440	0.1 μA	MBA He^2+^ implantation	[42]
0.1	−2	1549	0.1 nA	MBA Si^+^ implantation	[43]
8 × 10^−6^	−0,1	1550	-	IPA Cu/p-Si Schottky barrier	[44]
0.08 × 10^−3^	−1	1550	10 nA	IPA Cu/p-Si Schottky barrier	[45]
0.5–0.8	−5	1550	2.5 nA/mm	MBA Si^+^ implantation	[46,47]
50 × 10^−3^	−0.5	1330	120 μA/cm^2^	MBA Laser irradiation in presence of SF_6_	[48]
36 × 10^−3^	−11	1575	0.12 μA	SSA	[49]
0.25 × 10^−3^	−15	1541.5	2.5 nA	SSA-ring resonator	[50]
6 × 10^−3^	−3	1550	15 pA	TPA Photonic crystal resonators	[51]
2 × 10^−6^	1	1300	-	TPA Hemispherical structure	[52]
0.18	−6	1550	5 μA	IPA Al-porous Si Schottky barrier	[53]
35 × 10^−3^	−0.5	1550	120 μA/cm^2^	MBA in presence of SF_6_	[54]
